# Bioadhesive Hyaluronic Acid/Dopamine Hydrogels for Vascular Applications Prepared by Initiator-Free Crosslinking

**DOI:** 10.3390/ijms23105706

**Published:** 2022-05-20

**Authors:** Tamara Melnik, Senda Ben Ameur, Nasreddine Kanfar, Laurent Vinet, Florence Delie, Olivier Jordan

**Affiliations:** 1 Institute of Pharmaceutical Sciences of Western Switzerland, University of Geneva, Rue Michel-Servet 1, 1211 Geneva, Switzerland; tamara.morgoeva@unige.ch (T.M.); senda.ben-ameur@etu.unige.ch (S.B.A.); nasreddine.kanfar@unige.ch (N.K.); laurent.vinet@unige.ch (L.V.); florence.delie@unige.ch (F.D.); 2 School of Pharmaceutical Sciences, University of Geneva, Rue Michel-Servet 1, 1211 Geneva, Switzerland; 3 Service of Vascular Surgery, Department of Heart and Vessels, University Hospital, Rue du Bugnon 36, 1011 Lausanne, Switzerland

**Keywords:** hydrogel, hyaluronic acid, bioadhesion, mussel inspired polymers, initiator-free crosslinking, perivascular administration, atorvastatin

## Abstract

Intimal hyperplasia, a vascular pathology characterized by vessel wall thickening, is implicated in vein graft failures. For efficient prevention, a biodegradable drug delivery system should be applied externally to the graft for an extended time. Finding a gel suitable for such a system is challenging. We have synthesized HA-Dopamine conjugates (HA-Dop) with several degrees of substitution (DS) and used two crosslinking methods: initiator-free crosslinking by basic pH shift or commonly used crosslinking by a strong oxidizer, sodium periodate. The rheological properties, bioadhesion to vascular tissue, cytocompatibility with fibroblasts have been compared for both methods. Our results suggest that initiator-free crosslinking provides HA-Dop gels with more adequate properties with regards to vascular application than crosslinking by strong oxidizer. We have also established the cytocompatibility of the initiator-free crosslinked HA-Dop gels and the cytotoxicity of dopamine-sodium periodate combinations. Furthermore, we have incorporated a drug with anti-restenotic effect in perivascular application, atorvastatin, into the gel, which showed adequate release profile for intimal hyperplasia prevention. The oxidizer-free formulation with improved bioadhesion holds promise as an efficient and safe drug delivery system for vascular applications.

## 1. Introduction

Intimal hyperplasia (IH) is a pathophysiological process affecting vessels after a surgical injury, e.g., bypass grafting. It causes restenosis and graft failure with grave consequences for the patients [1]. Currently in the clinics, the attempt to prevent IH is by systemic drug administration, or drug coated stents in case of minor percutaneous interventions. However, for the vein grafts, only systemic regimen is available. The dose that can be administered systemically is limited due to side effects, and may not provide the required amount of the active pharmacological ingredient at the injury site, resulting in up to 40% of vein graft failure [2]. Perivascular administration, locally at the adventitia has been efficient to inhibit IH, with promising results observed with application of statins or anti-inflammatory drugs [3,4,5], reviewed in [6]. To date, there are no clinically approved formulations for targeted local perivascular prevention of graft failure after bypass surgery [7,8]. Therefore, a perivascular drug delivery system (DDS) for IH prevention is a much-needed alternative to the existing systemic and endovascular approaches.

As the surgeon will apply the DDS around the vessel during the open surgery, it should conform to the vessel shape and be easily spread on its surface. Ideally, the DDS has to be biodegraded once the drug is released. Offering versatile physical properties, hydrogels are promising candidates for such kind of DDS. Hydrogels can be loaded with a free drug for a rapid release and microparticles can be suspended as drug reservoir for long-term release [3].

Hydrogels are commonly used in numerous biomedical applications; they have been employed in reconstructive surgery, wound healing, as depots for drug delivery, and topical sustained-release systems. Thanks to its biocompatibility and biodegradability, hyaluronic acid (HA) is frequently used for this purpose, as reviewed in [9,10,11,12]. HA can be grafted with various moieties to allow crosslinking, or to confer certain properties, such as bioadhesion. HA conjugation with catechols, inspired by marine mussels, allows to increase bioadhesion properties [13,14,15,16,17], reviewed in [18]. Dopamine is commonly used to endow HA with adhesive properties, and to allow efficient crosslinking through oxidizing the dopamine groups by sodium periodate. However, the gels obtained via such crosslinking of HA-dopamine (HA-Dop) are too rigid to conform to vessel surface with, typically, a storage modulus (G′) of around 500 Pa at 4.5% degree of substitution (DS) with 2% polymer concentration and an equal stoichiometric ratio of sodium periodate to catechol [13]. For the application to tissues and organs such as blood vessels, the storage modulus of the gel obtained under these experimental conditions is too high and the gel too brittle to spread evenly on the application site. Furthermore, cytocompatibility concerns may be raised with 59% cells surviving after contact with HA-catechol [19].

To circumvent these limitations, we describe herein a pH-induced crosslinking of HA-Dop hydrogels with the purpose of tailoring their properties for perivascular application. We hypothesized that, avoiding strong oxidizers such as sodium periodate, HA-Dop gels would possess an improved safety profile, sparing the tissues from oxidative stress. Additionally, slow, pH-induced crosslinking should improve the elasticity and applicability to living tissues, as well as bioadhesion, which would allow the use of these gels as a drug delivery depot. The rheological properties, adhesion to vascular tissues, and cytocompatibility with fibroblasts were investigated. Furthermore, cytotoxicity of the combination of dopamine and sodium periodate was put into question and assessed, raising the need to seek other crosslinking mechanisms for mussel-inspired hydrogels. Lastly, the release of an antirestenotic drug, atorvastatin (ATV) from the NaOH-crosslinked HA-Dop gel has been evaluated.

## 2. Results

### 2.1. Synthesis and Characterization of HA-Dop

**Figure 1:**
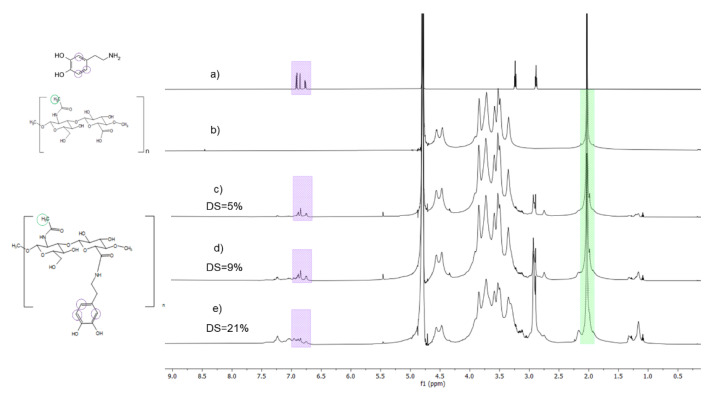
^1^H-NMR spectra of (**a**) dopamine, (**b**) hyaluronic acid (HA), (**c**–**e**) HA-dop with different DS (acetyl peak outlined in green, dopamine in purple).

The weight-average molecular weight of HA measured by size-exclusion chromatography was 190 kDa and the polydispersity index was 1.1 (Supplementary Table S1). To conjugate the dopamine to HA, an amidation reaction, mediated by a carbodiimide reagent (*N*-(3-dimethylaminopropyl)-N′-ethylcarbodiimide hydrochloride (EDC)/*N*-hydroxysulfosuccinimide sodium (sNHS) was performed as described by Shin et al. [13]. By changing the relative reactant quantities different DS were obtained (Table 1). Dopamine conjugation was confirmed by ^1^H-NMR spectra (Figure 1). Characteristic peak of acetyl group belonging to HA backbone was observed at 2 ppm, while the peaks of aromatic protons belonging to the catechol were observed at δ = 6.7–7.0 ppm (Figure 1). UV spectrophotometry-based DS were consistent with ^1^H NMR. In both calculations, the DS was determined per dimeric unit of HA (carboxyl group). Fourier-transform infrared spectroscopy (FTIR) spectra confirmed the structure of HA; however, the amide bond peak (1610 cm^−1^) coincides with the acetyl peak of HA (Supplementary Figure S1).

Further experiments were performed using the batches with low, medium, and high target DS (5%, 9%, and 21% according to NMR quantification).

### 2.2. Crosslinking of HA-Dop with Either Sodium Periodate or Sodium Hydroxide

**Figure 2:**
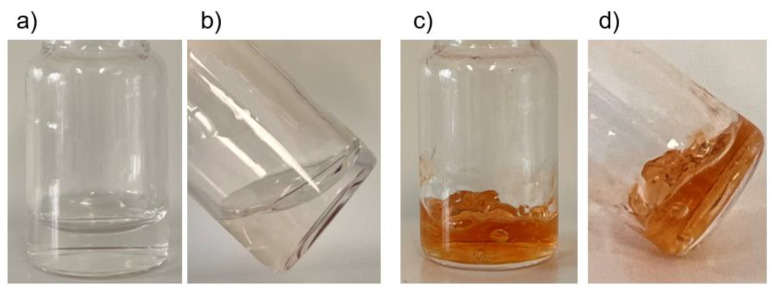
Macroscopic images of (**a**,**b**) HA-Dop, DS 5% before adding NaIO_4_. (**c**,**d**) HA-Dop, DS 5% 1 min after adding NaIO_4_.

Sodium periodate was added into solubilized HA-Dop at 1:2 molar ratio to dopamine, leading to periodate-crosslinked HA-Dop gels (further designated as NaIO_4_-CL gels). The crosslinking was rapid (<1 min), observed through a change of color to yellow and then brownish-red, and visible change in rheological properties (Figure 2).

**Figure 3:**
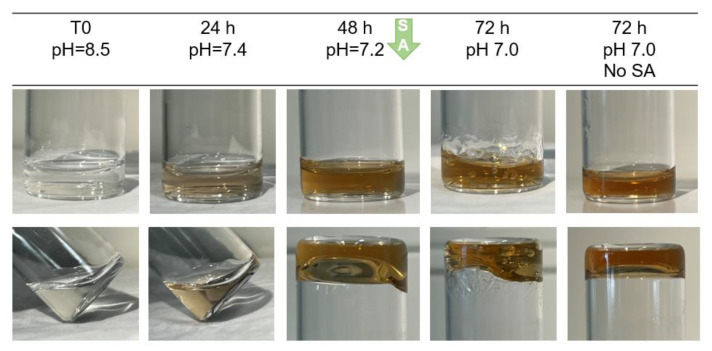
Macroscopic images showing the progressive change of color of HA-Dop (DS 5%) after basification with NaOH. At 48 h, the crosslinking was quenched by the addition of SA, and 24 h later, the gel color remained unchanged. The gel without addition of SA slightly intensified and reddened, suggesting that SA was effective to quench the crosslinking reaction.

When using sodium hydroxide, we observed a slower, progressive change of color over 48 h. At this point, sodium ascorbate (SA) was added, after which, the color remained constant, further confirmed by the UV absorbance (Supplementary Figure S2) at 420 nm (characteristic of quinones [20]). The obtained crosslinked gels (Figure 3) will be further designated as NaOH-CL gels.

**Figure 4:**
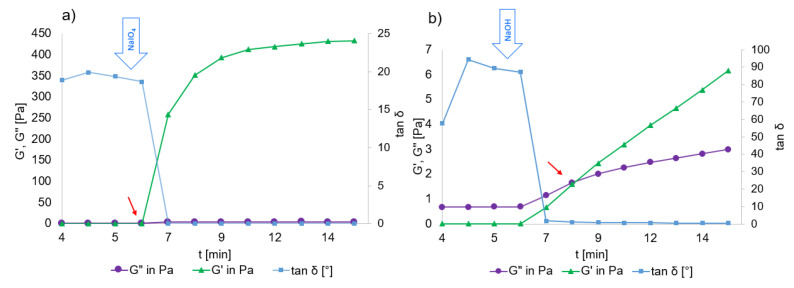
Rheological characterization of the initial crosslinking kinetics of HA-Dop (DS 9%) by NaIO_4_ (**a**) and by NaOH (**b**) in oscillation mode with a constant frequency of 1 Hz. The cross-linker was added at 5.5 min after the start of the measurement. Red arrows indicate gelation points (crossing of G′ with G′ and decrease of tan δ to below 1).

The effects of both crosslinking methods were assessed by oscillatory rheology. The cross-linkers were added after a 5.5 min conditioning step. Figure 4 shows the gel points (G′ = G″, tan δ = 1).

Figure 4 shows the evolution of G′, G″ and subsequently, tan δ as a result of crosslinking with NaIO_4_ and NaOH. In case of NaIO_4_, G′ starts to increase immediately upon addition (from 0.04 Pa before to 259.30 Pa 1 min after addition) and continued to increase, while tan δ decreased to 0.01. NaOH crosslinking was slower and the G′ increased from 0.01 Pa to 0.66 Pa at the first measurement point, with a slow continuous increase. In both cases, G″ remained relatively low before and after crosslinking, indicating that the viscous modulus of the hydrogel was maintained. The gelation points were at 30 s and 150 s after addition of NaIO_4_ and NaOH, respectively, as indicated by red arrows in Figure 4.

### 2.3. Rheological Properties of HA-Dop Crosslinked Gels

**Figure 5:**
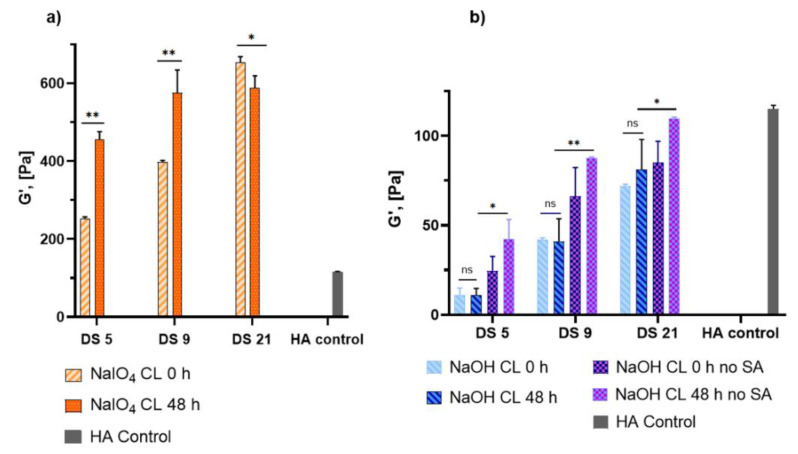
Evolution of the storage modulus (G′) of the NaIO_4_-CL and NaOH-CL gels at 0 and 48 h after crosslinking initiation (oscillation at 1 Hz). (**a**): G′ of NaIO_4_-crosslinked gels at the 3 DS. (**b**): G′ of NaOH-crosslinked gels with and without addition of SA. Control HA (Belotero Intense^®^) is given as a reference. * *p* ≤ 0.01; ** *p* ≤ 0.0001; ns: not significant.

To assess the rheological properties immediately after crosslinking, as well as to monitor their change over a longer period, the gels were subjected to oscillatory rheological measurements at time of crosslinking (0 h) and 48 h later (Figure 5).

The G′ values of the NaIO_4_-CL gels are all above 200 Pa right after crosslinking induction (G′0) and change dramatically 48 h after (G′48). There are no significant changes in G′0 and G′48 for NaOH-CL gels containing SA (*p* > 0.98), whatever the DS. In contrast, the gels without SA differ at G′48 at all DS from those containing SA.

### 2.4. Ex Vivo Adhesion to Porcine Aorta Tissues

**Figure 6:**
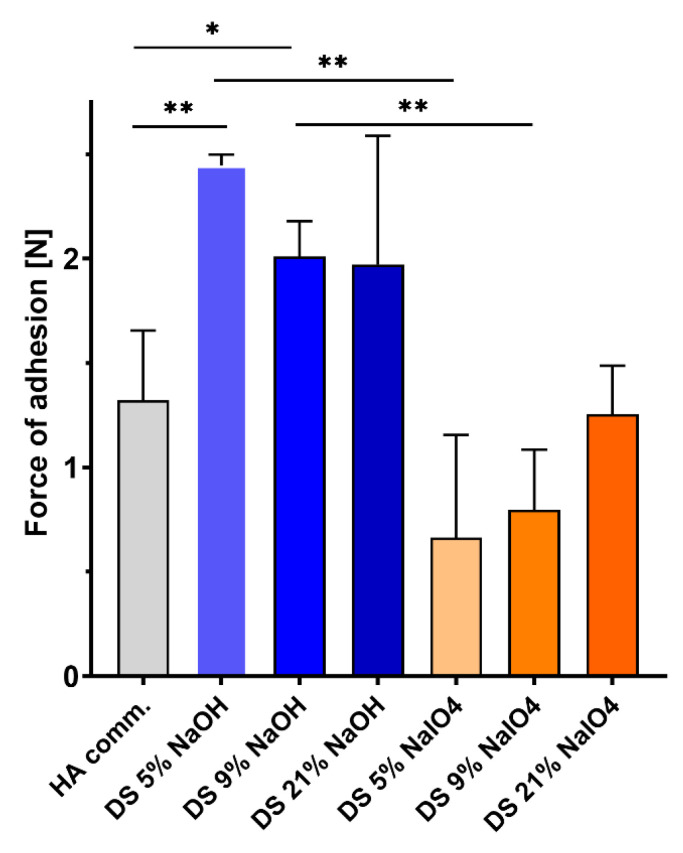
Adhesive properties of HA-Dop gels. Statistical analysis: unpaired *t*-tests. * *p* = 0.04, ** *p* = 0.003, *n* = 3.

To compare the adhesion of both types of gels and a control, non-functionalized crosslinked HA, the gels were brought in contact with porcine aorta tissues, and the maximum force of adhesion was measured. The adhesive properties of the gels are presented in Figure 6.

NaOH-CL gels exhibited higher bioadhesion than the control HA for all DS. NaOH-CL gels demonstrated superior bioadhesion compared to NaIO_4_-CL for all DS, with significant differences for DS 5% and 9%. Work of adhesion values are in line with these results and are presented in Supplemenary Figure S3. The force of adhesion of the NaIO_4_ crosslinked gels slightly increased with the DS, although there is no statistical significance when the 3 NaIO_4_-CL gels are compared among each other, and none of them is significantly different from the control. The gel with the highest dopamine content has similar adhesive properties to the control HA, while the DS 5% and 9% gels are less adhesive.

### 2.5. In Vitro Cytocompatibility with Fibroblast Cells

**Figure 7:**
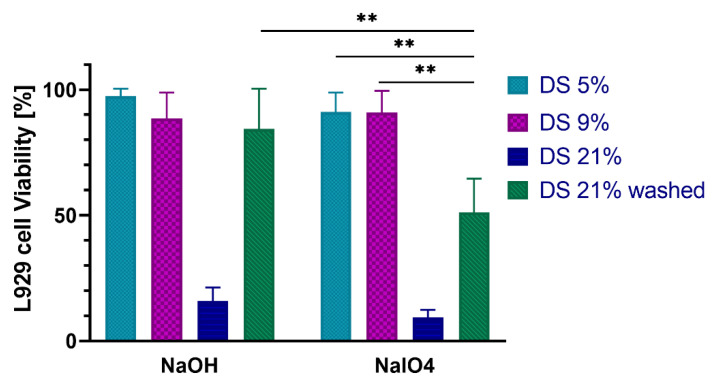
Viability of L929 cells after 24 h incubation with NaOH and NaIO_4_ crosslinked gel extracts. *n* = 3, error bars = SD; ** *p* < 0.002.

The results of L929 mouse fibroblasts viability after incubation with the gel extracts are presented in Figure 7. The gels with DS 5% and 9% were not toxic when crosslinked with either method, while both methods have caused toxicity of the DS 21% gels. An extensive washing step (48 h, 6 × media changes) helped to reduce the toxicity of the DS 21% NaOH-CL, while the NaIO_4_-CL DS 21% gel remained toxic even after washings.

**Figure 8:**
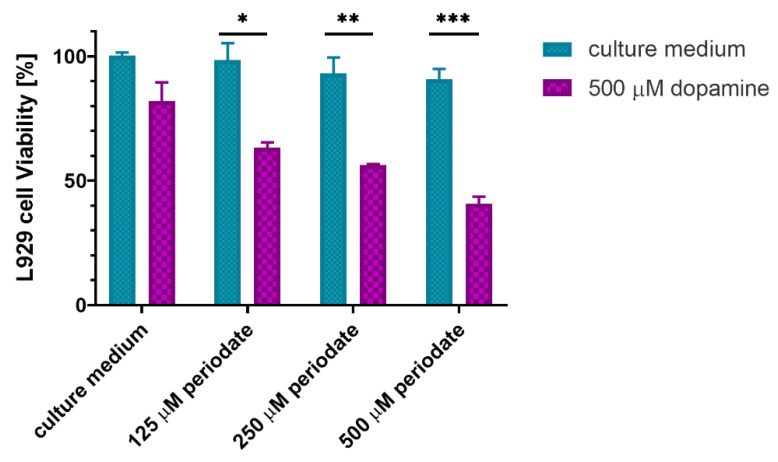
L929 cell viability after 24 h incubation with combinations of dopamine and sodium periodate. *n* = 3, error bars = SD. * *p*  = 0.03, ** *p*  =  0.003, *** *p*  <  0.0001.

To investigate the potential causes of gel toxicity, the sodium periodate crosslinked gel at the highest DS was incubated for 24 h in distilled water (dH_2_O) to determine the remaining dopamine concentration. The absorbance at 280 nm and referring the value to the calibration curve (Figure S4) indicated a 500 μM concentration of dopamine in the extract. The fibroblasts were exposed to combinations of dopamine at this concentration and sodium periodate at different ratios of dopamine to periodate (Figure 8).

Neither dopamine alone, nor periodate alone at any of the concentrations were toxic to the cells. However, when dopamine was combined with periodate, cell viability dropped significantly.

### 2.6. In Vitro Release of Atorvastatin from the HA-Dop NaOH-CL Gel

**Figure 9:**
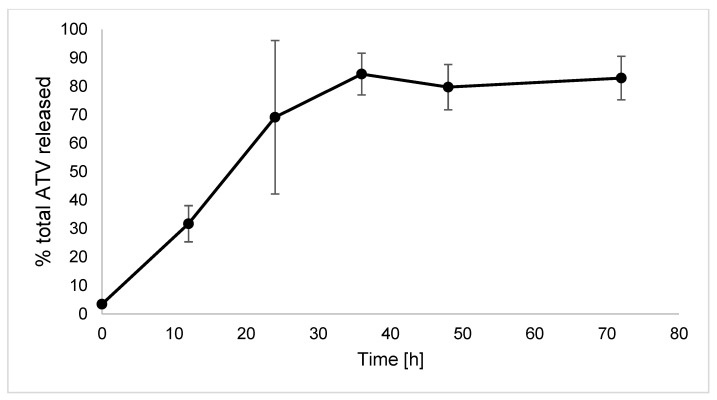
Cumulative release of atorvastatin from the HA-Dop NaOH-CL gel. *n* = 3, error bars = SD.

Figure 9 presents the ATV release kinetic from the HA-Dop NaOH-CL gel. Approximately 80% of the total drug (5 mg) is released by 36 h, after which the curve reaches a plateau. The analysis of the drug remaining in the gel after 72 h has shown 22.64% ± 4.01% of the total ATV.

## 3. Discussion

In this study, we aimed at developing a hydrogel possessing the adequate rheological and adhesive properties for a perivascular application, as well as validated cytocompatibility. We have synthesized batches with 3 DS of dopamine as quantified by ^1^H NMR and confirmed by UV and FTIR spectra (Figure 1, Table 1 and Figure S1). On FTIR spectrum, similarly to Lih et al., and Hong et al., the amidation peak at 1610 cm^−1^ is coinciding with the C=O group of HA [15,21]. Targeting a low, medium, and high DS, we have obtained the conjugates by changing the molar equivalents of the reactants. We have selected the molecular weight in the range of 130 to 300 kDa based on several publications on catechol conjugation to HA [13,17,22]. Some reports show that for efficient carboxylic group activation and amine coupling, an excess of EDC should be used, compared to sNHS and the amine [23,24]. Our results confirm that the DS increases with the amount of EDC equivalents used.

Figure 2 and Figure 3 illustrate the effect of crosslinking by two methods under basic pH using or not using a strong oxidizer, NaIO_4_. The alkaline pH is required to favor oxidative reactions by deprotonating catechol’s OH- groups [25]. The crosslinking occurs through quinone or semiquinone intermediates. When using NaIO_4_, the intermediates are formed very fast, hence the crosslinking occurs in a matter of seconds. In case of NaOH crosslinking, the oxidation occurs slowly by the oxygen contained in dH_2_O and in the sample’s headspace. Within the first 24 h, NaOH was consumed and the pH returned to neutral (Figure 3). The addition of SA allowed to avoid further crosslinking beyond this point through its anti-oxidative action and inhibition of dopamine-dopamine reaction [20].

Crosslinking kinetics, as investigated by oscillatory rheology, demonstrated that NaIO_4_ crosslinking occurs almost instantly, G′ increasing over 100-fold and reaching a plateau within 2 min (Figure 4). In case of NaOH crosslinking, the effect is milder with slower kinetics. The G′ increases slightly and continuously, without reaching a plateau. Time to gelation also differs; in case of NaIO_4_, it takes 30 s and NaOH crosslinking takes 150 s for tan δ to descend to 1.

The rheological properties of the NaIO_4_-crosslinked gels, shown in Figure 5 are in line with the reports of Shin et al. and Hong et al. [13,15]. Shin et al. used 1:1 periodate:dopamine molar equivalents at 2% polymer concentration, while Hong et al. used an excess of periodate with 1.5:1 ratio, final polymer concentration not reported. By using 1:2 periodate:dopamine at 2% concentration, we were aiming to reduce the crosslinking density, and indeed, obtained slightly lower values compared to these reports. However, for the purposes of perivascular application, these values are still too high, and the gel is too stiff for an easy application on a vessel. The gels with storage modulus (G′) over 200 Pa and tan δ below 0.1 are not suitable to conform to any shape other than that of the container they were molded in; when force is applied, they break, rather than spread. Due to rapid crosslinking obtained by NaIO_4_, the fast reaction hindered homogeneous crosslinked network formation. When adding NaIO_4_ drop-wise while stirring the gel, we observed polymer-dense and polymer-poor areas, which were not mixing well. Supplementary Figure S5 shows the G′ and tan δ at HA concentrations ranging from 0.5% to 1.5%, which are also not suitable for perivascular application. Additional disadvantage of sodium periodate crosslinking for the envisioned application is the continuous increase of G′ at 48 h, which shows that the gels continue to cross-link.

These results suggest that the crosslinking density initiated by sodium hydroxide in the absence of NaIO_4_ is lower compared to sodium periodate crosslinking density. This allows a better handling of the gel when applying to vascular surfaces and conformation to the vessel shape. The quenching with SA allowed to “freeze” the state of the gel as there was no difference between the gels at 0 h and 48 h.

To the best of our knowledge, no reports have evaluated the bioadhesion of HA-Dop crosslinked only by NaOH to the vascular tissues. The data presented in Figure 6 demonstrates that all the NaOH-CL gels had better adhesion to the aorta tissues than the control HA soon after application to the tissues. They were also more adhesive than their NaIO_4_ crosslinked counterparts, although in the case of the gel with DS 21% this effect was not statistically significant. A long-term adhesion assay could provide more insights into the dopamine-tissue interactions, although maintaining viable ex vivo tissue on the long-term is challenging.

The aim of dopamine conjugation to HA is to provide adhesive properties to the gel. However, when crosslinking is performed with sodium periodate, the adhesion is not improved compared to the non-functionalized crosslinked HA. Oxidation with NaIO_4_, creates a cohesive gel, resulting in reduced adhesion [15]. In the absence of a strong oxidizer, the gel is less cohesive and has more freedom to react with the nucleophiles (such as, −NH2, −SH) contained in the biological tissues [17,26].

The adhesivity of NaOH-CL gels appeared to reduce (although not significantly) when dopamine DS increases (Figure 6). It could be explained by the influence of the rheological properties on the adhesion measurement. The lower is G′, the more liquid is the gel resulting in a better spreading on the probe and on the tissue. This provides more tissue-gel interface and may result in higher adhesion. This known bias is intrinsic to the measurement of bioadhesion in highly deformable gels.

Overall, the NaOH-induced crosslinking has created an elastic gel with appropriate rheological properties and improved tissue adhesion compared to the commercially available, non-functionalized HA.

Investigating a critical issue of biomaterials, cytotoxicity, we selected the mouse fibroblast cell line as a model to mimic the cells contacting the formulation in the tunica adventitia of the blood vessels [27]. The viability results (Figure 7) support the safety of all the gels at the DS 5% and 9%. At a high DS of 21%, however, cytotoxicity appeared, which could be suppressed by washing only for the NaOH-CL gels.

The mechanism of this toxicity is attributed to the oxidized forms of dopamine, the quinones, which, depending on the chemical structure, can have both cytotoxic, and cytoprotective effects [27]. The toxicity of the NaOH-CL gel with DS 21% was alleviated by washing, which hints at the reversibility of the OH- induced oxidation. However, for NaIO_4_-CL gel, the toxicity remained an issue after the washing.

We hypothesized that this effect might be attributed to the combination of the dopamine with sodium periodate, and the particular form of quinone it generates. We compared the viability of the L929 cells in medium with or without dopamine (at the concentration found in the extract of DS 21% NaIO_4_-CL gel, 500 μM) when associated or not with different concentrations of sodium periodate typically used for crosslinking (1:1; 1:2 and 1:4) (Figure 8). The data shows that the combinations are toxic, while the agents on their own are not. This points out that sodium periodate may not be an optimal crosslinking agent for HA-Dop gels, due to the reactive form of quinone it generates.

For the incorporation of the anti-restenotic drug, ATV, we selected the gel-candidate that was suited best for perivascular application according to the results obtained herein, HA-Dop with 9% DS, crosslinked by NaOH. D-α-Tocopheryl polyethylene glycol 1000 succinate (TPGS) played the role of a surfactant allowing to disperse poorly water-soluble ATV in the hydrogel. A drug release study demonstrated a first-order release until most of the drug was released at 36 h time point. This fast release is typical for HA gels and comparable to the three-day release previously reported with ATV [3]. However, the mass balance at 72 h revealed that 23% of ATV was still in the gel, in contrast to conventional HA gels. This might be explained by inter-molecular interactions between ATV-Ca^2+^ and HA-Dop, possibly through ionic interaction, such as in iron-crosslinked HA-Dop gels [28] or the interaction of aromatic rings of ATV and dopamine. Such a gel would thus be adequate to deliver high load (several mg) of ATV over the first days to initiate tissue healing and provide initial anti-inflammatory effect, whereas a sustained activity could be provided by the incorporation of drug reservoirs such as biodegradable microparticles.

## 4. Materials and Methods

### 4.1. Materials

Sodium hyaluronate (molecular weight range 130–300 kDa) was obtained from Contipro Biotech Ltd., Dolní Dobrouč, Czech Republic. Dopamine hydrochloride, sodium periodate, sodium hydroxide, hydrochloric acid (HCl) 37%, EDC, sNHS, TPGS, D_2_O, Phosphate-Buffered Saline (PBS) 1× and PBS 10×, were purchased from Sigma-Aldrich. SA was obtained from HÄNSELER AG, Germany. Atorvastatin calcium was obtained from Chemos GmbH&Co.KG (Altdorf, Germany). L929 cells (mouse fibroblasts) were purchased from ECACC (Salisbury, UK); Dulbecco’s Modified Eagle’s Medium (DMEM)—high glucose, 10% fetal calf serum from Eurobio, 500 U/mL penicillin and 500 μL/mL of Steptomycin (Pen Strep) and 0,25% Trypsine-EDTA (1×) were purchased from Life Technologies Corporation (Paisley, UK); cell proliferation reagent 2-(4-iodophenyl)-3-(4-nitrophenyl)-5-(2,4-disulfophenyl)-2H-tetrazolium monosodium salt (WST-1) from Roche Applied Science, Basel, Switzerland. Control HA (2.55% Belotero Intense^®^) was obtained from Merz Aesthetics, Allschwil, Switzerland.

### 4.2. Methods

#### 4.2.1. HA Molecular Weight Measurement

The weight-averaged molecular weight (Mw) was obtained by size exclusion chromatography (SEC) coupled to a multi angle laser light scattering and refractive index detector (AF2000, Postnova, Landsberg am Lech, Germany); the data were treated in Postnova AF2000 software.

#### 4.2.2. Synthesis of HA-Dop

HA-Dop was synthesized as previously described. Briefly, 250 mg of HA were solubilized into 25 mL dH_2_O under magnetic stirring. Various molar equivalents of EDC and sNHS were added simultaneously, and 30 min later dopamine was added. The molar equivalents of each compound are reported in Table 1. The pH of the reaction mixture was controlled and maintained at 5.5 by adding HCl or NaOH dropwise. The reaction was left under stirring in the dark overnight. The reaction mix was subsequently transferred into dialysis membrane (Spectrum Labs, San Francisco, CA, USA, MWCO 6–8 kDa), immersed into PBS at pH 6, with 3 changes of the PBS during 24 h, and then the membrane was placed into dH_2_O, with 3 changes of the medium. After dialyses, the mixture was freeze-dried to get a white sponge-like solid structure and stored in an air-tight container at +4 °C until use.

#### 4.2.3. DS Determination by NMR Spectroscopy

The DS was determined by ^1^H NMR on a Bruker Avance Neo 600 MHz NMR spectrometer (Bruker BioSpin, Rheinstetten, Germany) supplied with a QCI 5 mm Cryoprobe and a SampleJet automated sample changer. Freeze-dried HA-Dop samples were solubilized in D_2_O at around 3 mg/mL under stirring for 2 h. Spectra were treated with MestReNova (Mnova) software V10.0 (Mestrelab, Santiago de Compostela, Spain). D_2_O solvent peak was used as reference peak and chemical shifts were reported as parts per million (ppm). DS was calculated from the ratio of the peaks of the three dopamine aromatic protons at δ = 6.7–7.0 ppm, to the peak of HA N-acetyl group (three protons) at δ = 1.9–2.0 ppm.

#### 4.2.4. DS Determination by UV-Vis Spectroscopy

Calibration standards were prepared by consecutive dilutions of dopamine in dH_2_O (1.56, 3.13, 6.25, 12.5, 25, 50 μg/mL) and were read out at 280 nm on a microplate reader (Synergy Mx, Biotek, Winooski, VT, USA) in triplicates. Freeze-dried HA-Dop samples were solubilized in dH_2_O at a concentration of 0.5 mg/mL and read out in triplicates. The absorbance at 280 nm was referred to the calibration curve (Supplementary Figure S4) and the DS was calculated.

#### 4.2.5. FTIR

HA, Dopamine and HA-Dop conjugates’ spectra have been analysed with a Perkin Elmer Spectrum 65 spectrophotometer in the range between 600 and 4000 cm^−1^ with a universal ATR Sampling Accessory in solid phase.

#### 4.2.6. HA-Dop Crosslinking with Sodium Periodate

A solution of HA-Dop in PBS 1× was prepared at 2.2% (*w*/*v*). 100 μL of an aqueous periodate solution was then added into 900 μL of the HA-Dop solution at pH = 8.5 to have a final molar ratio of 1:2 (periodate:dopamine, as measured by NMR). The final concentration of HA-Dop was 2% (*w*/*v*). The gels were used immediately upon crosslinking for rheological and adhesion experiments. The samples for cytotoxicity tests were freeze-dried immediately upon crosslinking.

#### 4.2.7. HA-Dop Crosslinking with Sodium Hydroxide

A solution of 2% (*w*/*v*) HA-Dop in dH_2_O was prepared under nitrogen blanket. Once dissolved, NaOH 1 M was added until pH = 8.5 and left to cross-link for 48 h, at room temperature. pH was measured every 24 h. To quench the crosslinking, SA solubilized in water at 10% of dopamine mass was added to the gel. Further measurements and tests were performed precisely 24 h after SA addition. The samples for cytotoxicity tests were freeze-dried 24 h after the addition of SA.

#### 4.2.8. Rheological Measurements

Viscoelastic properties of the gels were determined with a HAAKE Mars Rheometer™ (ThermoFisher Scientific, Waltham, MA, USA) with a cone-plate C35 2°/Ti rotor. Approximately 500 μL of gel samples were loaded on the measuring plate and storage modulus (G′) and loss modulus (G′’) were recorded at 25 °C, at a frequency sweep of 0.1 to 4.68 Hz. Tan δ was calculated as the ratio of G′’ to G′. Gel rheological properties were compared to a crosslinked, control HA (Belotero Intense^®^) that was shown in previous studies to possess adequate viscoelasticity for an easy application at the vascular site [3].

#### 4.2.9. Adhesion to Porcine Aorta

The assay was performed on porcine aorta purchased from a local slaughterhouse using the “Gel Mucoadhesion Probe” of the Texture Analyser TA.XTPlusC (Stable Microsystems Ltd., Surrey, UK). The aorta was cut into squares of approximately 8 cm^2^. A specimen was placed into the mucoadhesion rig and conditioned in a beaker of PBS heated to 37 °C. Then, the probe carrying the gel (450 μL) was brought into contact with the aorta and a force of 4.9 N was applied for 120 s. Three repetitions were done for each test. A photo of the setup is provided in Supplementary Figure S6.

#### 4.2.10. Gel Extracts Preparation and Cytotoxicity Tests

Gel extracts were prepared by dispersing 10 mg of freeze-dried crosslinked HA-Dop into 4 mL of fetal calf serum (FCS) supplemented DMEM, incubated in a rotary shaker at 37 °C at 55 rpm for 24 h. Extracts were obtained by collecting the supernatant, which was then filtered through a 0.22 μm filter. For the high DS gels, a washing step was added: after crosslinking, the gels were placed into a dialysis membrane with MWCO 6–8 kDa and washed for 48 h, with 6 changes of medium: 3× PBS and 3× dH_2_O. After washing, gels were freeze-dried for 48 h and extraction was carried out as described above. To test the toxicity of dopamine/periodate combinations, solutions were prepared in culture medium.

Cytotoxicity of the samples was determined on L929 mouse fibroblast cell line cultured in 75 cm^2^ culture flasks (Corning Incorporated, Corning, NY, USA) in DMEM (high glucose supplemented with 10% FCS, 500 U/mL penicillin and 500 μL/mL of streptomycin), kept in a humidified atmosphere with 5% CO_2_ and 95% air until reaching 90 to 100% confluence. The cells were used at passages 2–34. At confluence, cells were harvested using 2 mL trypsin for 5 to 7 min until most cells were detached. The trypsin was neutralized with 8 mL supplemented DMEM. The cells were seeded in 96-well cell culture plates with flat bottom and low evaporation lid at a density of 10,000 cells per well and cultivated for 24 h. The medium was removed and replaced with 100 μL of the extracts for 24 h. Cell viability was measured using a colorimetric assay for 96-well plates with WST-1 reagent according to the supplier’s recommendations. One hour after adding WST-1, absorbance was measured at 450 nm and 690 nm on a microplate reader (Synergy Mx, Biotek, Winooski, VT, USA) and absorbance values were subtracted. Each plate contained positive (culture medium) and negative (SDS 0.1% *w*/*v*) controls in triplicates. Cytotoxicity of the compounds was expressed as percentage of cell viability compared to positive controls. The results are presented as triplicates (3 plates, each one containing triplicate wells).

#### 4.2.11. Determination of Dopamine Concentration in the Gel Extract

Dopamine concentration in the extracts was determined by UV-VIS spectrophotometry on gel extracts, obtained as described in Section 4.2.10, using dH_2_O instead of DMEM. The absorbance was read out at 280 nm. Dopamine calibration curve was prepared in dH_2_O.

#### 4.2.12. Incorporation of ATV into the Gel, In Vitro Drug Release Study

To incorporate ATV into the gel, 5 mg of ATV were suspended in 1 mL of 0.1% TPGS. HA-Dop, DS 9%, was dissolved in the ATV-TPGS suspension to obtain a 2% (*w*/*v*) solution. The crosslinking followed the steps described in Section 4.2.7.

For drug release study, 100 μL of HA-Dop-ATV gel were put into 4.5 mL release medium (PBS 1×/SDS 0.1%) allowing sink conditions in triplicates. The 3 vials were placed in a rotary incubator (80 rpm) at 37 °C. At predetermined time intervals, 0.5 mL samples were taken and replaced with fresh medium. Samples were filtered through 0.22 μm PVDF syringe filters and stored refrigerated until testing.

For ATV quantification, Waters Acquity™ Ultraperformance LC (Milford, MA, USA) was used. The column used was Ethylene Bridged Hybrid (BEH) C18, (Waters, Ireland), maintained at 30 °C. The analytical run lasted 5 min with a flow rate of 0.3 mL/min, at the gradient described in Supplementary Table S2. The calibration curve was made by dissolving ATV in 30% ethanol solution and diluting consecutively in (PBS 1×/SDS 0.1%/TPGS 0.1%) to obtain the range from 0.78 to 50 μg/mL (Supplementary Figure S7). Eluates were monitored at 245 nm.

For mass balance, at the end of the experiment, the gels were removed from the release medium, lyophilized, and left in methanol for 2 h to extract the remaining ATV. The supernatant was then collected, diluted 5× in mobile phase and analyzed.

#### 4.2.13. Statistical Analyses

An unpaired Student’s *t*-test was performed for the comparison of average values from two datasets. For datasets with multiple variables comparisons, a two-way repeated measures ANOVA with a post-hoc Dunnett test were performed. A *p* value lower than 0.05 was considered statistically significant.

## 5. Conclusions

In this study, we have elaborated a crosslinking method for HA-Dop, avoiding strong oxidizers, and providing slower oxidation kinetics compared to sodium periodate. This allows to control the crosslinking and develop a gel suitable for perivascular applications. Indeed, despite extensive studies on HA-Dop gels, the available crosslinking technique proved inadequate for direct application to vascular tissue.

Considering its potential clinical applications, the oxidizer-free DDS would also be a more acceptable option from a regulatory point of view. For future perspectives, the gel should be tested in vivo to ascertain feasibility of perivascular application and pharmacological efficiency.

## Figures and Tables

**Table 1:** Molar equivalents of the reactants relative to HA carboxyl and mean DS obtained over *n* = 3 batches of HA-Dop synthesis (*n* = 3).

Target DS	EDC	sNHS	Dopamine	HA-Dop DS (NMR)	HA-Dop DS (UV)
Low	2.0	1.2	1.2	5.7 ± 0.6	5.4 ± 0.9
Medium	3.0	1.3	1.3	9 ± 1	9.4 ± 2.0
High	5.0	1.5	1.5	19 ± 2	18 ± 5

## Data Availability

The data presented in this study are available on request from the corresponding author.

## Supplementary Materials

The following supporting information can be downloaded at: https://www.mdpi.com/article/10.3390/ijms23105706/s1.
