# Supplementary material for: Bioadhesive Hyaluronic Acid/Dopamine Hydrogels for Vascular Applications Prepared by Initiator-Free Crosslinking
Source: Int J Mol Sci. 2022 May 20;23(10):5706. doi: 10.3390/ijms23105706 (PMC9146728; doi:10.3390/ijms23105706)
Supplement: Supplementary file 1 [file ijms-23-05706-s001.zip › ijms-1726213-supplementary.pdf]

# Bioadhesive hyaluronic acid/dopamine hydrogels for vascular applications prepared by initiator-free crosslinking

**Tamara Melnik, Senda Ben Ameur, Nasreddine Kanfar, Laurent Vinet, Florence Delie and Olivier Jordan**

**Supplementary materials.**

**Table S1.** Size exclusion chromatography-multiangle light scattering measurement of $M_n$, $M_w$ and PDI of the raw HA

| | AVG (n=3) | SD |
|---|---|---|
| $M_n$ [g/mol] | 1.67E+05 | 8.7E+03 |
| $M_w$ [g/mol] | 1.90E+05 | 2.5E+03 |
| PDI | 1.1 | 0.0 |

**Table S2**. Gradient of mobile phases for U-HPLC detection of atorvastatin

| *Time (minutes)* | *A, 10 mM Ammonium formate + 0.1 formic acid, [%]* | *B, acetonitrile + 0.1 formic acid, [%]* |
|---|---|---|
| 0.0 | 60 | 40 |
| 3.2 | 20 | 80 |
| 3.3 | 60 | 40 |
| 5.0 | 60 | 40 |

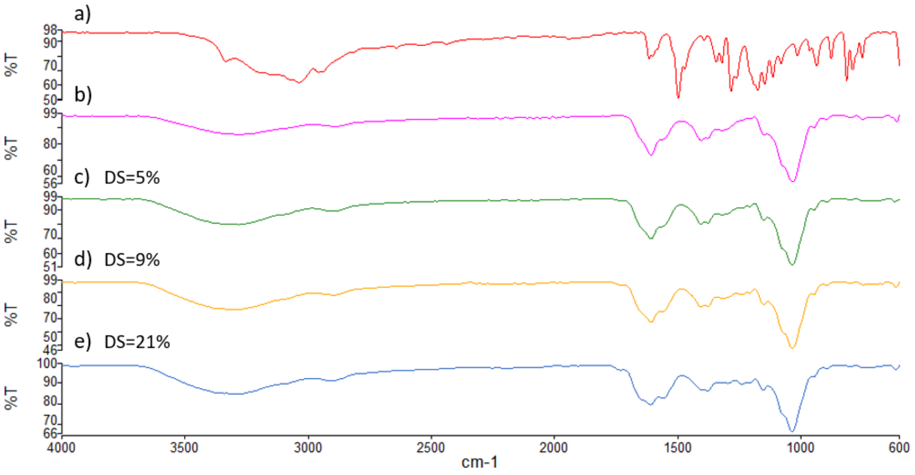


**Figure S1.** FTIR spectra of a) dopamine, b) hyaluronic acid, c-e) HA-dopamine with different degrees of substitution. The amide bond, confirming the successful conjugation of dopamine to HA is seen at 1610 $cm^{-1}$, coinciding with C=O group of HA. The wide band at 3280 $cm^{-1}$ represents the hydroxyl groups of HA. The stretching vibrations belonging to dopamine's C-H at 2920 $cm^{-1}$ and C=C at 1520 $cm^{-1}$, are also present in the HA-Dop, however, to a less extent, as it is less concentrated than pure dopamine.

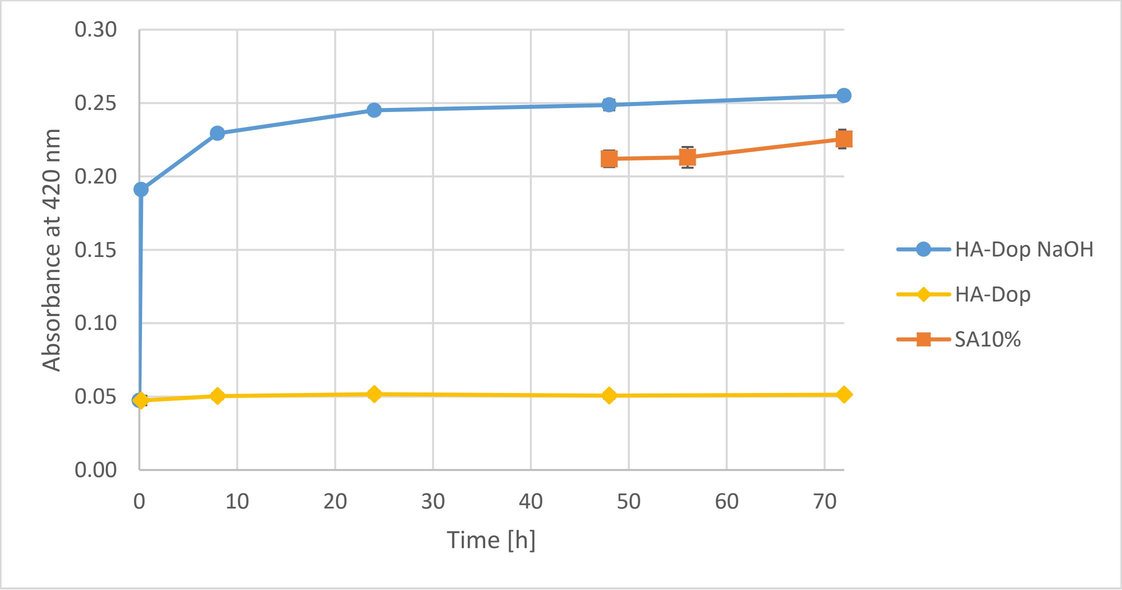


**Figure S2.** UV absorbance at 420 nm (quinone) of HA-Dop (DS 9%), with addition of NaOH and sodium ascorbate (SA) at 48 h, n=3.

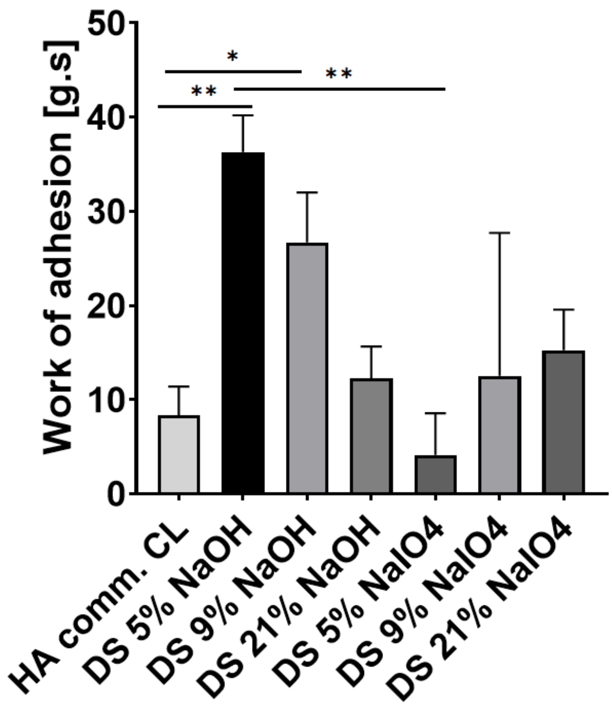


**Figure S3**. Work of adhesion of different gels measured on Texture Analyzer with porcine aorta tissues. * $p \leq 0.01$; ** $p \leq 0.0001$.

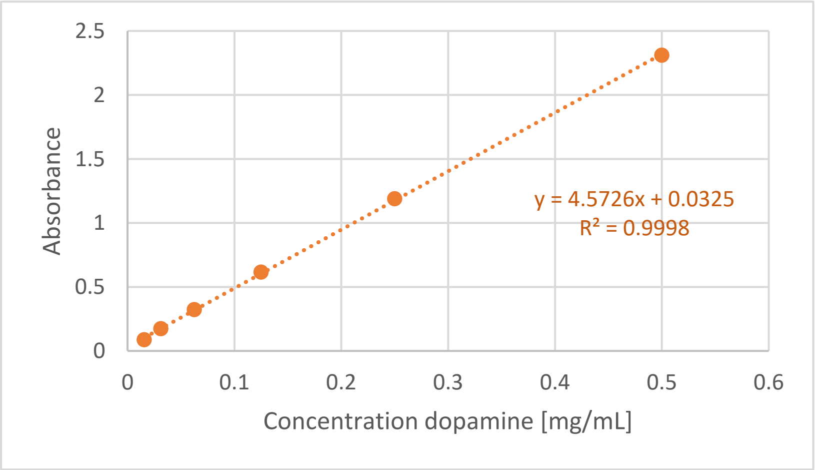


**Figure S4**. Dopamine calibration curve in $dH_2O$ at 280 nm

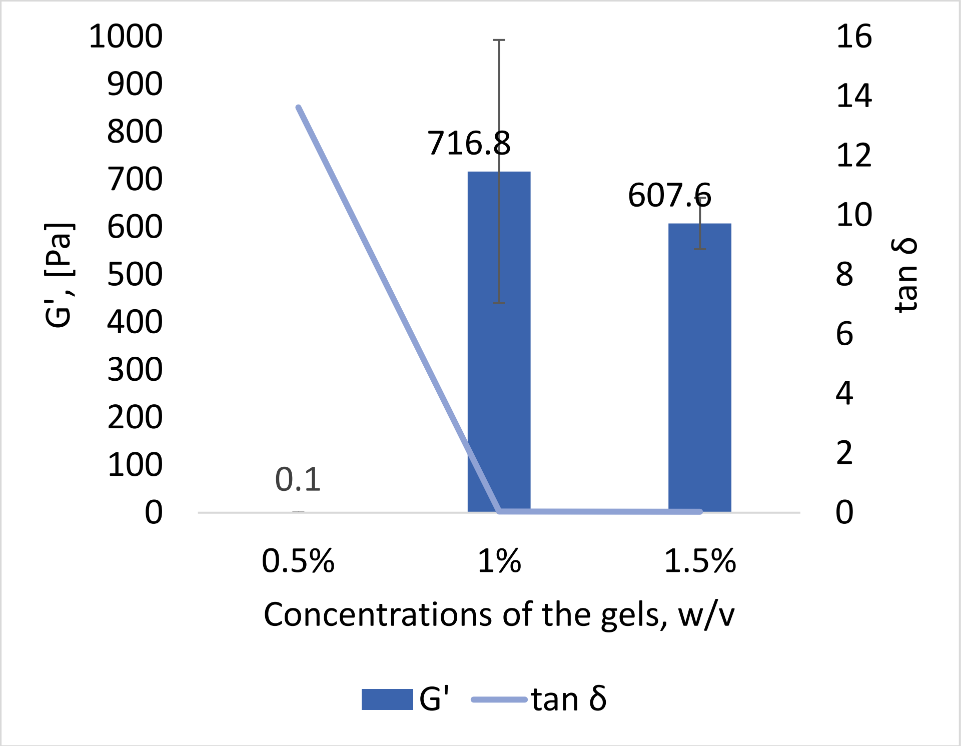


**Figure S5.** Evolution of G′ and tan δ of cross-linked by 1:2 $NaIO_4$ gels (DS 9%) at different concentrations from 0.5% to 1.5%, n = 3, error bars = SD. The 0.5% is a liquid solution, while 1.0% and 1.5% are too rigid and inhomogenous for perivascular application.

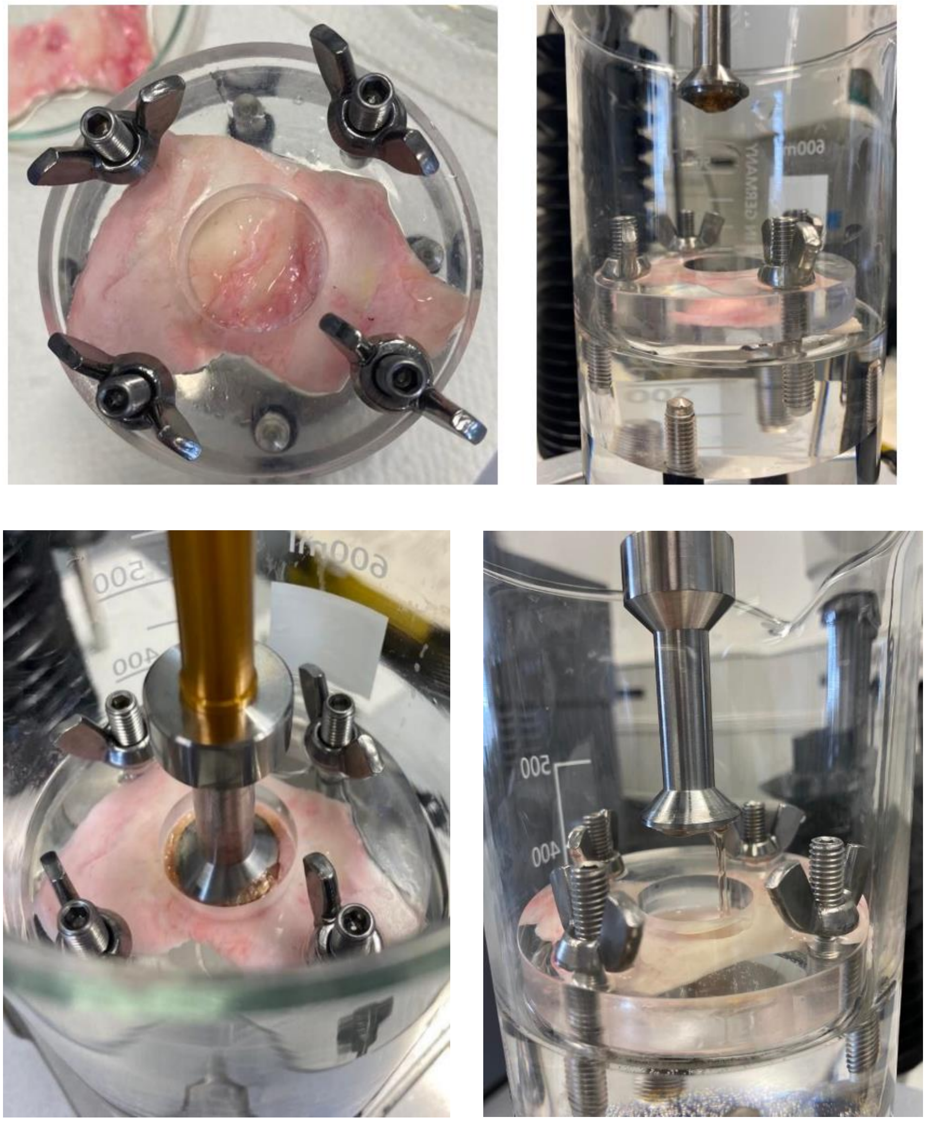

**Figure S6**. Bioadhesion testing set-up, views from different angles.

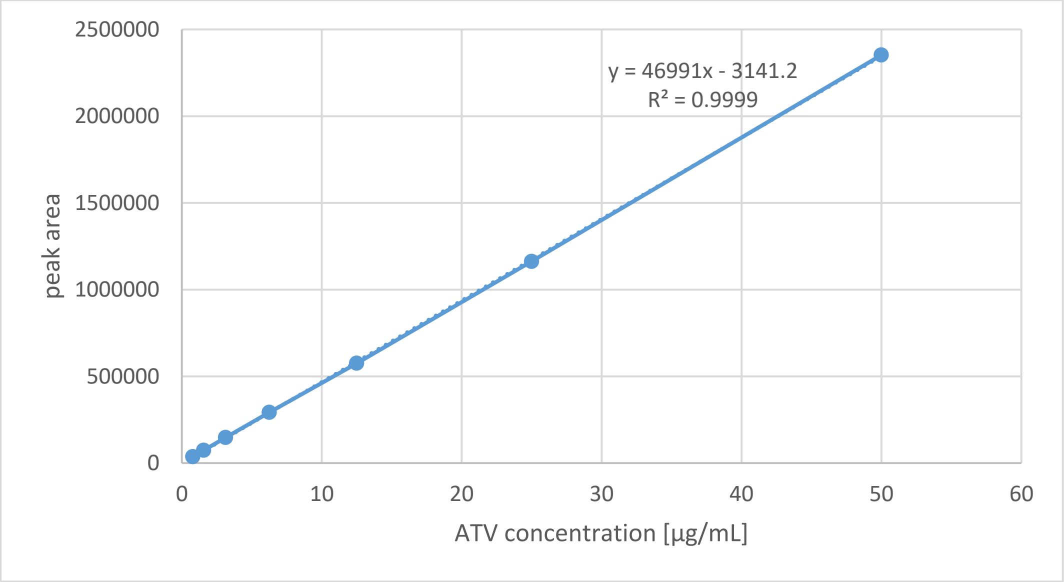


**Figure S7.** Calibration curve of atorvastatin obtained by monitoring eluates of calibration standards at 245 nm
